# Light—Vision

**Published:** 1882-10

**Authors:** 


### EDITORIAL.

### Light—Vision.

Nowhere are good light and correct vision more important than in the examination of and operations upon the natural teeth. In many places it is impossible to have clear and unobscured light. In any place, even the most favorable, there will be sometimes a hazy, or smoky, or a cloudy atmosphere. Some localities are subject to these disabilities in a much greater degree than others. Large cities, and indeed small ones, that are compactly built have defective light, being most of the time somewhat obscured by smoke, vapors and buildings, and under such circumstances the light is exceedingly variable.

Marshy localities will have a hazy atmosphere, much more than the higher, drier and hilly places. The state of the atmosphere and the surroundings are important elements in estimating for good light. Light from the north is preferable to that from any other quarter, chiefly because it is more uniform and more steady; light from other quarters is often used, but is never so satisfactory. It is true that screens and shades may be used to cut off an excess of light, but these do not meet the fluctuations that are so frequent in light from east, south or west. Having a good light, the next point is its management, or rather, making it most available and efficient. In every instance the rays of light should come upon the point of observation as directly as possible. It is not practicable to place all the localities in the mouth requiring an operation in the same position, in respect to the rays of light, at least as they are usually admitted. But arrangements in buildings are sometimes so made as to make the light available all the way from the zenith to the horizon; this is accomplished by inclined windows and sky-lights. The latter gives special advantage in operations upon the inferior teeth; but there are disadvantages that perhaps overbalance. The light from the zenith is not as desirable as that from the horizon to a point midway to the zenith: and, again, the cross-rays from a skylight and a vertical window are very objectionable; if both are present it is almost a necessity to obscure the one while the other is being used. In operations upon the lower teeth the reflector, either in the mouth or out of it, and especially with a little condensing power, can be made to more than compensate for the skylight.

The best arrangement is a good vertical window with pure crown glass extending to eighteen to twenty inches of the floor, three feet wide, and the height just sufficient for the patient, when seated and the head in position for an operation upon the upper molars, to see just above the midway-point between the horizon and the zenith. The window should have two equal sections of inside blinds, one above and one below; each section should be divided into two, of three leaves each, hinged together, this arrangement puts the amount of light entering the window under perfect control. There should be no side window near the chair; every chair, however, should have a rotary movement. The light in an operating room should be considerably less than that outside.

The management of light near and in the mouth is a matter of importance and one requiring considerable tact. Several sizes and forms of reflectors should be at hand, nearly all of which should have more or less of condensing and magnifying power. By means of these light can be thrown into obscure places, and increased by condensation as may be desired. Magnifiers of various sizes and power should always be at hand, these in many instances may be used for light condensers; for this purpose, however, they are only available when the light falls directly upon the point of observation. Of the various arrangements for magnifiers there is, perhaps, none more common or more effective than properly adjusted eye-glasses. Still, it is questionable whether those who have good, unimparied eye-sight, should accustom themselves to glasses constantly while operating, but the occasional use of the glass in some form as a magnifier is almost a matter of necessity.

The importance and value of good light and its proper management in dental practice is strongly emphasized, when we consider that the wonderful revelations made by the microscope, is by good light and its skillful management, through the use of the most perfect machinery and appliances.

By the various aids, to vision, that are available, much may be learned of the structure, condition, and defects of the various tissues with which we have to do; and not only this, but the operations performed will be more thoroughly accomplished under a stronger and more penetrating vision than can be obtained by the unaided eye. Material defects in dentine and enamel, especially the latter, often exist that can only be made apparent by concentrated light and a glass of good definition. The things that ought to be seen and correctly estimated in every tooth that is filled, are,—*First*, The structural quality of the tissue. *Second*, The congenital defects, as shown in fissures, imperfect or open tracts through the enamel, atrophy, spots, pits or grooves in the enamel. *Third*, The accidental defects, such as fractures, checks, and change of color. *Fourth*, The minute roughness and irregularities upon the surfaces of the teeth, and in cavities that are to be filled, especially in the borders. *Fifth*, The particular appearance of decay as exhibited in the different varieties, and the various stages of each.

The faculty of observation varies greatly in different persons; some see everything, others comparatively little. Some see finely, others coarsely. The impressions made by observation upon some are permanent, upon others of very short duration. The faculty of close and effective observation is an important one to the dentist, one in which many are deficient. It is one, however, susceptible of improvement by proper cultivation, and every one should see to it that this part of his education is not neglected. It should also be kept in mind that in this particular each one is his own teacher.

